# Fractal Spiking Neural Network Scheme for EEG-Based Emotion Recognition

**DOI:** 10.1109/JTEHM.2023.3320132

**Published:** 2023-09-28

**Authors:** Wei Li, Cheng Fang, Zhihao Zhu, Chuyi Chen, Aiguo Song

**Affiliations:** School of Instrument Science and EngineeringSoutheast University Nanjing Jiangsu 210096 China

**Keywords:** Electroencephalogram, fractal spiking neural network, inverted drop-path, emotion recognition

## Abstract

Electroencephalogram (EEG)-based emotion recognition is of great significance for aiding in clinical diagnosis, treatment, nursing and rehabilitation. Current research on this issue mainly focuses on utilizing various network architectures with different types of neurons to exploit the temporal, spectral, or spatial information from EEG for classification. However, most studies fail to take full advantage of the useful Temporal-Spectral-Spatial (TSS) information of EEG signals. In this paper, we propose a novel and effective Fractal Spike Neural Network (Fractal-SNN) scheme, which can exploit the multi-scale TSS information from EEG, for emotion recognition. Our designed Fractal-SNN block in the proposed scheme approximately simulates the biological neural connection structures based on spiking neurons and a new fractal rule, allowing for the extraction of discriminative multi-scale TSS features from the signals. Our designed training technique, inverted drop-path, can enhance the generalization ability of the Fractal-SNN scheme. Sufficient experiments on four public benchmark databases, DREAMER, DEAP, SEED-IV and MPED, under the subject-dependent protocols demonstrate the superiority of the proposed scheme over the related advanced methods. In summary, the proposed scheme provides a promising solution for EEG-based emotion recognition.

## I. Introduction

Recognizing emotions automatically and accurately not only can aid in diagnosis and treatment of mental disorders, but also can assist in nursing and healthcare during postoperative rehabilitation. Generally, signals available for emotion recognition can be categorized into external and internal ones. External signals mainly include expression, speech, behavior, and so on. Internal signals primarily refer to physiological signals, such as Electroencephalogram (EEG) and Electrocardiogram (ECG). Exterior signals have the merits of being intuitive and easily accessible. However, exterior signals may be unreliable if individuals deliberately conceal their emotions. In contrast, interior signals are difficult to feign or counterfeit, which can provide reliable evidence for emotion recognition. EEG, as one kind of interior signal, usually has different morphologies under different human emotional states [1]. Besides, EEG is a measurement of neural activity, which is closely related to human emotions. Therefore, EEG is a highly reliable physiological cue for emotion recognition.

In recent years, there have been notable advancements in the field of EEG-based emotion recognition. Cui et al. [2] put forward a Regional-Asymmetric Convolutional Neural Network (RACNN), which is capable of extracting spatio-temporal information, for emotion recognition. Li et al. [3] brought forward a Bi-hemisphere Domain Adversarial Neural Network (BiDANN), which encompasses a global domain and two local domains of discriminators that work adversarially with a classifier, to learn discriminative EEG features for emotion classification. Wang et al. [4] put forth a hybrid Spatial-Temporal Feature Fusion Neural Network (STFFNN), which is composed of CNN, feedforward network and bidirectional Long Short-Term Memory (Bi-LSTM), to extract spatial-temporal features from EEG and integrate complementary information from these features to recognize emotions. Zhong et al. [5] brought forth a Regularized Graph Neural Network (RGNN), which effectively captures both local and global relationships between EEG channels, for recognizing emotions. Li et al. [6] put up a Bi-Stream MLP-SA Mixer (BiSMSM), which captures EEG features from spatial, temporal, local and global perspectives, for classifying emotions. Peng et al. [7] brought up a temporal self-attention mechanism and a channel self-attention mechanism, which can utilize the temporal and spatial information of EEG signals, to classify emotions.

In addition to the above research, some researchers have also used biologically-inspired neural networks to recognize emotions from EEG. Typically, Spiking Neural Network (SNN) employs spiking neurons with temporal dynamics to construct the network with robustness for spatio-temporal feature exploring and asynchronous event processing [8], [9], [10]. Existing research has already demonstrated the capacity of SNN to extract the spatio-temporal representation from EEG [11], [12], [13]. For instance, Kasabov [8] devised a unified computational framework based on an unsupervised 3D evolving SNN architecture, which is named as NeuCube; NeuCube allows for the integrative modeling and learning of various spatial- and spectral-temporal brain data. Luo et al. [11] extracted frequency features from EEG, then transformed the features into spike sequences through the step-coding rule, and finally employed Neucube to learn the discriminative representation from these features. Alzhrani et al. [12] presented a Brain-Inspired Spiking Neural Network (BISNN) architecture to classify emotional states, and to visualize the spatial-temporal relationships between the EEG channels in a 3D SNN model. Tan et al. [13] came up with a short-term emotion recognition framework, which can extract the spatio-temporal spiking pattern features from EEG in the trained SNN reservoir for classification.

In summary, many studies suggest that the Temporal-Spectral-Spatial (TSS) information in EEG is useful for emotion classification. Nevertheless, they only pay attention to such information at one single scale rather than the multiple scales, which may provide more assisting power for the issue of EEG-based emotion recognition. Considering this, in our paper, we propose a new and effective method, Fractal-SNN scheme, to exploit the multi-scale TSS information for the issue. In brief, the main contributions and innovations of this work are as follows:

• We propose a novel method, the Fractal-SNN scheme, which can effectively exploit the multi-scale TSS information in EEG for emotion recognition.
• We design an $F_{2}(\bullet)$ block based on $Axon$ and $Soma$ operations to approximately simulate biological neural connection structures. We generalize the $F_{2}(\bullet)$ block into the Fractal-SNN block $F_{c}(\bullet)$ via a new fractal rule.
• We devise a training technique, inverted drop-path, for $F_{c}(\bullet)$ block. Attributing to this technique, the block can learn different TSS information from EEG features based on different sub-networks in the training phase.
• Our experiments on the benchmark databases DREAMER, DEAP, SEED-IV and MPED sufficiently demonstrate the effectiveness of the Fractal-SNN scheme for EEG-based emotion recognition.

## II. Methods

**FIGURE 1.**
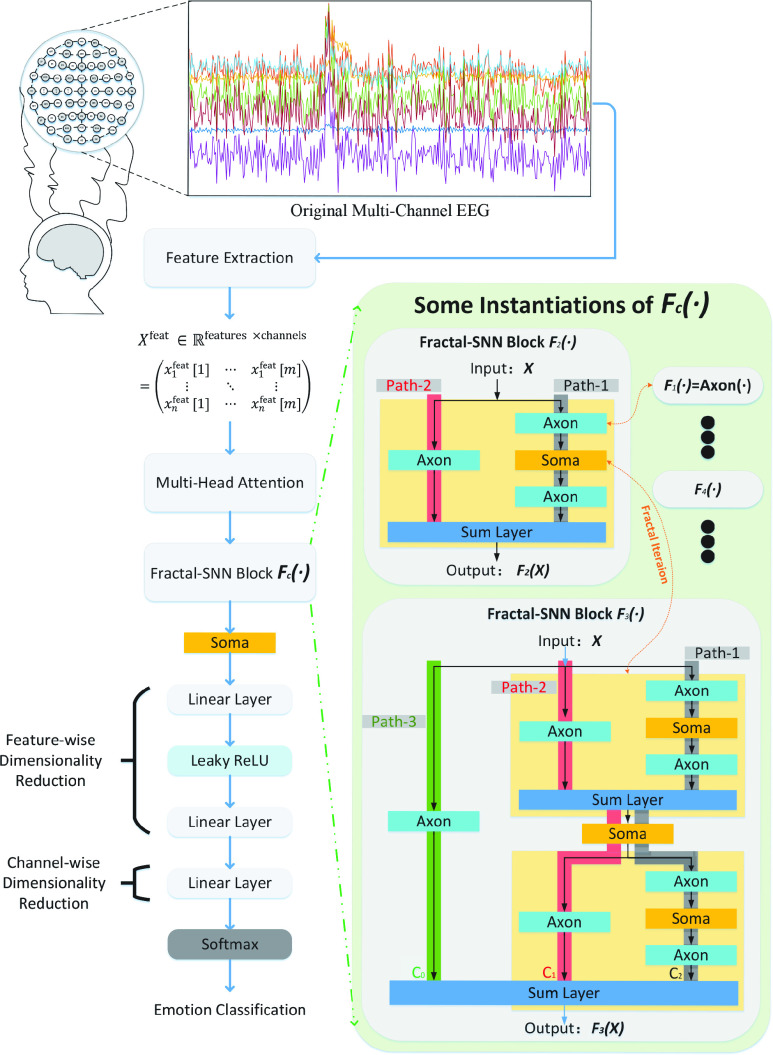
Framework of fractal spiking neural network (Fractal-SNN) scheme, which consists of five components: feature extractor, multi-head attention module, Fractal-SNN block, dimensionality reduction module and classifier. In this figure, Fractal-SNN block $F_{c}(\bullet)$ is generated by a new fractal expansion rule, which will be introduced in Section II-D.3. And, we show two specific examples of $F_{2}(\bullet)$ and $F_{3}(\bullet)$. To facilitate comprehension of the concept of “sub-networks with different path lengths” within the block, we distinguish the three paths in the $F_{3}(\bullet)$ by three different background colors (i.e., green, red, and gray). The operations (e.g., $Axon(\bullet)$ and $Soma(\bullet)$.) traversed by these three paths respectively comprise “three sub-networks with different path lengths”. Note that the paths shown in the figure are the specific cases, and the actual path depends on the inverted-drop path technique in the $Sum~Layer$, which will be introduced in Section II-D.4.

The whole framework of the Fractal-SNN scheme has been illustrated in Fig. 1. In brief, the procedures of the Fractal-SNN scheme are as follows: firstly, we extract the TSS feature matrix from multi-channel EEG signals; next, we input the feature matrix into the multi-head attention module to obtain the weighted feature matrix; then, we feed the weighted feature matrix into the Fractal-SNN block to acquire the high-level TSS feature matrix from different channels; afterward, we reduce the dimensions of the high-level feature matrix; finally, we utilize the softmax function for emotion classification.

### A. Feature Extraction

DREAMER [14] and DEAP [15] contain the raw EEG signals, while SEED-IV [16] and MPED [17] contain the EEG features. Thus, it is imperative to extract features from EEG in DREAMER and DEAP at first. Before extracting features, we preprocess the EEG signals in the following way: we select the signal intervals in the last 60 seconds of each EEG at first, and then process the signals with a 4~60 Hz bandpass filter; after that, we perform $z$–score normalization on the filtered signal data. We denote Preprocessed EEG (PE) as $X^{\textrm {PE}}\in \mathbb {R}^{s\times m}$, where $s$ is the sampling point number per channel of EEG, and $m$ is the number of EEG channels.

We extract features from the PE of each channel. For temporal-spectral features, we first segment the PE into EEG segments using a sliding window method with a window length of 1 second and a step size of 0.5 second. For each EEG segment, we extract Power Spectral Density (PSD) and Differential Entropy (DE) features in four frequency bands: $\theta $ band (4~8 Hz), $\alpha $ band (8~14 Hz), $\beta $ band (14~30 Hz) and $\gamma $ band (30~45 Hz). We concatenate the features of each segment from four frequency bands into the four long feature vectors according to the order of the original segments, respectively. Finally, the four feature vectors are further concatenated in the order of $\theta $, $\alpha $, $\beta $ and $\gamma $. After extracting the temporal-spectral features from all channels, $X^{\textrm {PE}}$ is converted into a feature matrix $X^{\textrm {feat}}\in \mathbb {R}^{n\times m}$ containing TSS information, where $n$ denotes the feature number per channel of EEG.

### B. Multi-Head Self-Attention Module

The multi-head self-attention module is widely used in the field of emotion recognition [6], [7]. Based on the self-attention mechanism, this module extracts features from sequence data and effectively captures dependencies within the data. In the Fractal-SNN scheme, we make use of the multi-head self-attention module to adaptively assign weights to the EEG features of each channel.

Let $X^{\textrm {feat}} = (x^{\textrm {feat}}[{1}]^{T}, \ldots, x^{\textrm {feat}}[i]^{T}, \ldots, x^{\textrm {feat}}[m]^{T})\in \mathbb {R}^{n\times m}$ be the EEG feature matrix, where $x^{\textrm {feat}}[i]^{T}=(x_{1}^{\textrm {feat}}[i],\ldots,x_{n}^{\textrm {feat}}[i])^{T}\in \mathbb {R}^{n}$ is the feature vector in channel $i$, $n$ denotes the feature length per channel of EEG, and $m$ denotes the number of EEG channels. Supposing that the multi-head self-attention module has $h$ heads, the feature vector $x^{\textrm {feat}}[i]^{T}$ will be divided into $h$ parts (i.e., $x^{\textrm {feat}}[i]^{T}=(x_{\alpha ^{1}}^{\textrm {feat}}[i], \ldots, x_{\alpha ^{h}}^{\textrm {feat}}[i])^{T}$). Then the input of attention head $\alpha $ is $x_{\alpha} ^{\textrm {feat}} [i]^{T}=(x_{\alpha _{1}} ^{\textrm {feat}} [i],\ldots,x_{\alpha _{n/h}}^{\textrm {feat}} [i])^{T}\in \mathbb {R}^{n/h}$, where $\alpha =\alpha ^{1},\ldots,\alpha ^{h}$. The output $x_{\alpha} [i]$ of each attention head is calculated by

\begin{equation*} x_{\alpha }[i]^{T} = f(q_{i\alpha },k_{i\alpha },v_{i\alpha }), \tag{1}\end{equation*}

where $q_{i\alpha } = W_{\alpha }^{(q)} x_{\alpha }^{\textrm {feat}}[i]^{T}$, $k_{i\alpha } = W_{\alpha }^{(k)} x_{\alpha }^{\textrm {feat}}[i]^{T}$ and $v_{i\alpha } =W_{\alpha }^{(v)} x_{\alpha }^{\textrm {feat}}[i]^{T}$; $W_{\alpha }^{(q)}, W_{\alpha }^{(k)}, W_{\alpha }^{(v)}\in \mathbb {R}^{n/h\times n/h}$ are the learnable weight matrices; $f(\bullet)$ represents the attention pooling, i.e., the scaled dot-product attention:

\begin{equation*} f(q_{i\alpha },k_{i\alpha },v_{i\alpha }) =\textrm {softmax}\left({\frac {q_{i\alpha }k_{i\alpha }^{T}}{\sqrt {n/h}}}\right) v_{i\alpha }. \tag{2}\end{equation*}

Then for channel $i$, the output of the multi-head self-attention module is $x[i]^{T} =W_{io}(x_{\alpha ^{1}}[i], \ldots, x_{\alpha ^{h}}[i])^{T} \in \mathbb {R}^{n}$, where $W_{io}\in \mathbb {R}^{n\times n}$ denotes the linear transformation matrix. Therefore, the output matrix after the input $X^{\textrm {feat}}$ passes through the multi-head self-attention module is $X = (x[{1}]^{T}, \ldots, x[i]^{T}, \ldots, x[m]^{T})\in \mathbb {R}^{n\times m}$.

### C. Spiking Neuron Model

For SNN, there are mainly two types of spiking encoding techniques: rate coding and temporal coding [18]. Rate coding, which is the representation of spike counts in a time window, only considers the statistics of spike activity. The SNN models based on rate coding are incompetent to learn the sequence structure information in the signals. However, in many fields including EEG-based emotion recognition, the spike timing and sequence structure of spike trains also contain the useful information [19]. Hence, we make use of temporal coding, which encodes the signals by precise spike timing, to represent the sequence structure information in EEG. Besides, we take advantage of Infinite Impulse Response formulated SNN (IIR-formulated SNN) [10] to capture the spatio-temporal sequence information in EEG signals. Dirac delta function is widely used to model the input into the neurons of SNN, because this function is simple and effective to represent the spike timing and the sequence structure of the input.

Let the input spike trains from the presynaptic neuron $b$ be a sequence of time-shifted Dirac delta functions: $x_{b} = \sum _{k}\delta (t-t_{kb}^{\textrm {fir}})$, in which $t_{kb}^{\textrm {fir}}$ denotes the $k^{\textrm {th}}$ spike arrival time from the presynaptic neuron $b$, and $y_{a}=\sum _{l}\delta (t-t_{la}^{\textrm {fir}})$ denotes the output spike trains from the neuron $a$. Thus, the SNN model is reformulated as the linear constant-coefficient difference equations:

\begin{align*} v_{a}[t] &= -\lambda r_{a}[t] + \sum _{b\in \Gamma _{a}}w_{ba}f_{b}[t], \tag{3}\\ r_{a}[t] &= e^{\frac {-1}{\tau _{r}}}r_{a}[t-1] + V_{\textrm {rest}} y_{a}[t], \tag{4}\\ f_{b}[t] &= \alpha _{1}f_{b}[t-1] + \alpha _{2}f_{b}[t-2] +\beta x_{b}[t], \tag{5}\end{align*}

where $v_{a}[t]$ is the membrane potential of the neuron $a$; $r_{a}[t]$ represents the reset filter of the neuron $a$; $f_{b}[t]$ represents the synapse filter $b$, which is a second order IIR filter; $w_{ab}$ is the learnable synaptic weight between synapse $a$ and neuron $b$; $x_{b}[t]$ denotes the $b^{\textrm {th}}$ input of synapse filter; $\alpha _{1} = e^{\frac {-1}{\tau _{m}}} + e^{\frac {-1}{\tau _{s}}}$, $\alpha _{2} = -e^{-\frac {\tau _{m} + \tau _{s}}{\tau _{m}\tau _{s}}}$, and $\beta = e^{\frac {-1}{\tau _{m}}} - e^{\frac {-1}{\tau _{s}}}$.

Let $Axon(\bullet)$ represent the operation of transmitting data to the next neuron. This operation can be simulated by the second-order exponential IIR filter in Eq. 5. Let $Soma(\bullet)$ represent the behavior of neurons updating the membrane potential and firing spikes. Specifically, the neuron’s membrane potential $v_{a}[t]$ is updated through $Soma(\bullet)$, as shown in Eq. 3–4. At the same time, if the membrane potential $v_{a}[t]$ in the neuron $a$ exceeds a certain threshold $V_{\textrm {thre}}$, $Soma(\bullet)$ will produce an output spike $y_{a}[t]$ as

**FIGURE 2.**
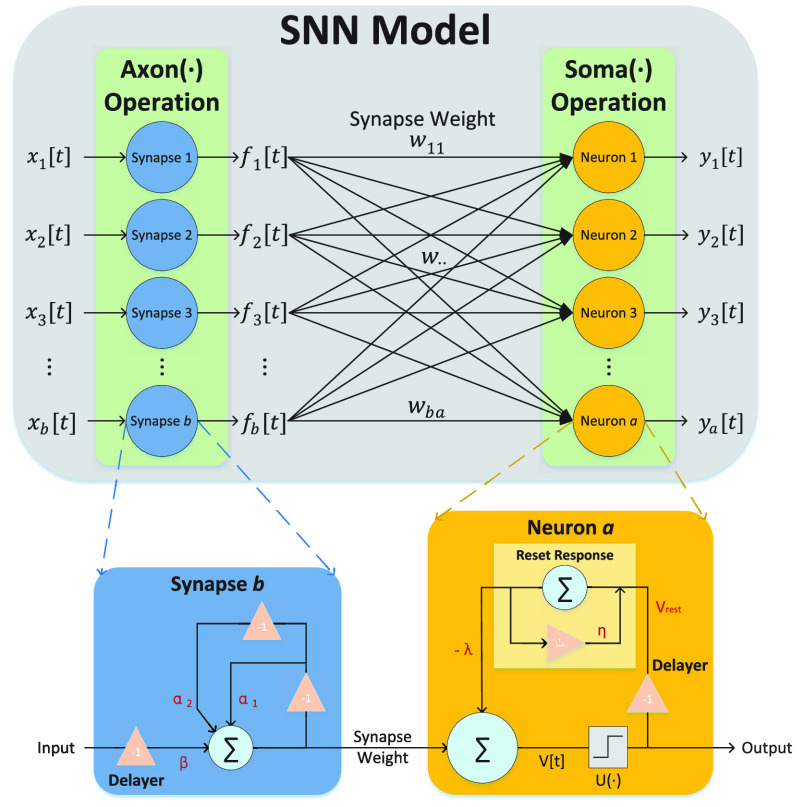
The SNN model constructed from infinite impulse response filters is divided into two sub-layers in this paper: $Axon$ and $Soma$. $Axon$ contains $b$ synapses, while $Soma$ contains $a$ neurons. There are connections between all synapses and neurons, and the connection strength is represented by the synaptic weight. For instance, the synaptic weight between synapse $b$ and neuron $a$ is denoted as $w_{ba}$. And, the calculation mechanism of synapses and neurons is also illustrated in the figure, where Delayer means delaying the sequence data by one unit.

\begin{equation*} y_{a}[t] = U(v_{a}[t] - V_{\textrm {thre}}), \tag{6}\end{equation*}

where $U(\bullet)$ represents the Heaviside step function. Therefore, the SNN model simulated by the IIR filters can be split into two operations: $Axon(\bullet)$ and $Soma(\bullet)$, as shown in Fig. 2

### D. Fractal Spiking Neural Network Block

Many studies have shown that the correlation between EEG channels is beneficial for emotion recognition [20], [21], [22]. Generally speaking, these methods involve the specific modules to capture the correlation information between EEG channels. Inspired by these methods, we design a block based on spiking neurons to exploit the multi-scale TSS information in EEG features across different channels.

To this end, we design an SNN model $F_{2}(\bullet)$ with two pathways and describe the forward propagation in $F_{2}(\bullet)$. Then, we integrate $F_{2}(\bullet)$ with the fractal structure to develop a more generalized Fractal-SNN block $F_{c}(\bullet)$, and also equip this block with the suitable training technique. Finally, we elucidate the learning mechanism of the Fractal-SNN block.

#### 1) Design Motivation

**FIGURE 3.**
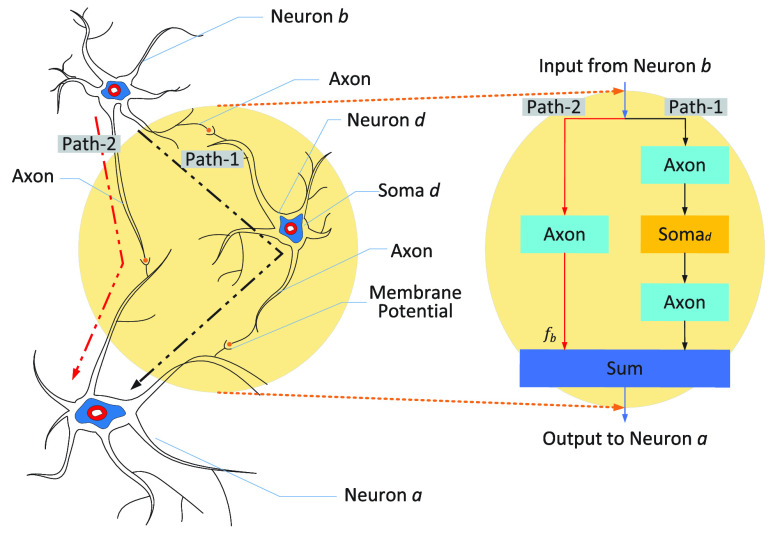
Neural analogue of $F_{2}(\bullet)$ block. The left part of the figure exhibits a connection structure consisting of three biological neurons, while the right part displays the modeling of this connection structure using two operations ( $Axon$ and $Soma$) in the spiking neuron. To better illustrate the relationship between the two parts of the figure, we provide an example in the right figure: each of $Axon$ and $Soma$ operations contains only one synapse and one neuron, just like the specific case of Fig. 2 where there is only one synaptic weight.

Advanced neural activities, such as cognition, learning, memory, and intelligence, rely on the cooperation and regulation of the brain components with different functions. Inspired by this biological mechanism, we use $Axon$ and $Soma$ operations to model different functional modules, as shown in Fig. 3. In some sense, the working mechanism of the designed SNN model $F_{2}(\bullet)$ block can be compared to the above biological mechanism: two sub-networks with different “lengths” of paths have different functional characteristics, but their outputs can be fused before being transmitted to the target neuron $a$.

#### 2) $F_{2}(\bullet)$ Block

**FIGURE 4.**
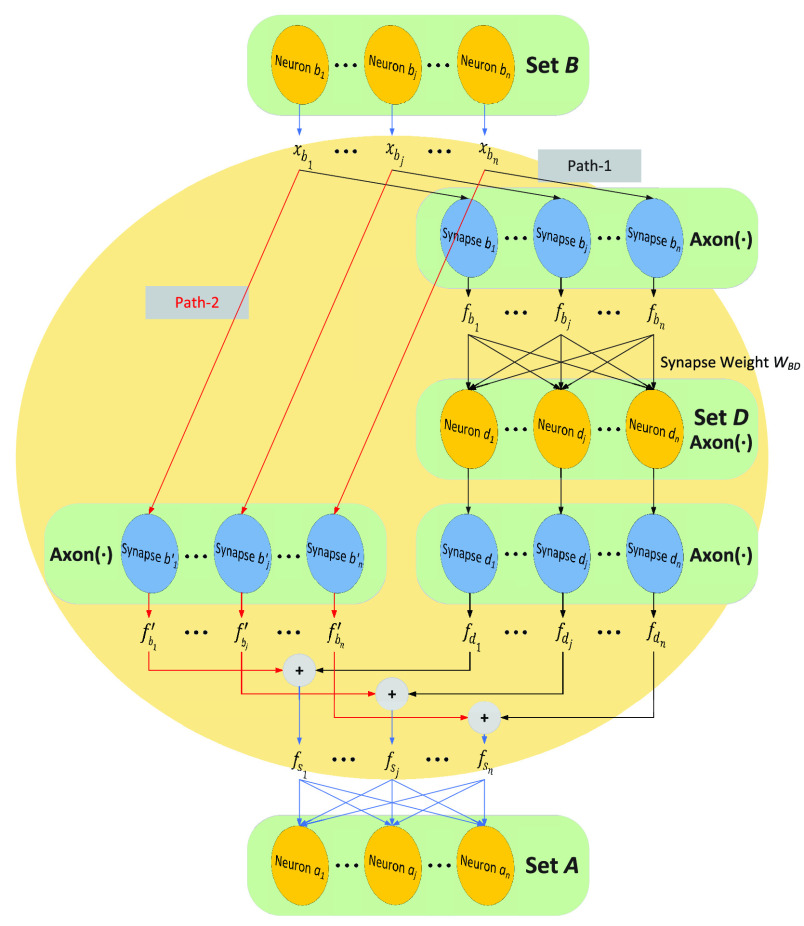
Forward propagation of $F_{2}(\bullet)$ block with $n$ synapses in $Axon$ and $n$ neurons in $Soma$. In the figure, the neurons of set $B$ can transmit signals to the neurons of set $A$ along two different paths, where Path-1 is represented by black arrow lines, and Path-2 is represented by red arrow lines.

Fig. 3 illustrates a neurobiological analogy, in which $Axon$ contains a synapse and $Soma$ contains a neuron. In the calculation process, each of $Axon$ and $Soma$ contains $n$ synapses and $n$ neurons, where $n$ is the number of features of each channel, as shown in Fig. 4. We denote the set containing neurons $b_{j}$ as $B$, the set containing neurons $d_{j}$ as $D$, and the set containing neurons $a_{j}$ as $A$, where $j=1,\ldots,n$. We use the output of set $B$ as the input of $F_{2}(\bullet)$ block, and use the input of set $A$ as the output of $F_{2}(\bullet)$ block.

The input of the $F_{2}(\bullet)$ block can be described as $X_{B}=(x_{b}[{1}]^{T}, \ldots, x_{b}[i]^{T}, \ldots, x_{b}[m]^{T})=(x_{b_{1}}, \ldots, x_{b_{j}}, \ldots, x_{b_{n}})^{T}\in \mathbb {R}^{n\times m}$, where $x_{b}[i]^{T}\in \mathbb {R}^{n}$ is a feature vector in channel $i$ and $x_{b_{j}}\in \mathbb {R}^{m}$ is a channel sequence formed by selecting the $j^{\textrm {th}}$ feature from each of the $m$ feature vectors. The input $X_{B}$ of the $F_{2}(\bullet)$ block is then transmitted along two paths, namely Path-1 and Path-2, to the neurons in set $A$.

For Path-1, the input $X_{B}$ passes through $Axon(\bullet)$. And the change in the synapse $b_{j}$ is expressed as

\begin{equation*} f_{b_{j}}[i] = \alpha _{1} f_{b_{j}}[i-1] + \alpha _{2} f_{b_{j}}[i-2] + \beta x_{b_{j}}[i], \tag{7}\end{equation*}

where $i=1, \ldots, m$, and $f_{b_{j}}[-1]$ and $f_{b_{j}}[{0}]$ are initialized as zeros. The output sequence of synapse $b_{j}$ is $f_{b_{j}}=(f_{b_{j}}[{1}], \ldots, f_{b_{j}}[i], \ldots, f_{b_{j}}[m])^{T}\in \mathbb {R}^{m}$. All synapses operate on their own input channel sequence according to Eq. 7. As a result, we obtain the output matrix $O_{B}=Axon(X_{B})=(f_{b_{1}}, \ldots, f_{b_{j}}, \ldots, f_{b_{n}})^{T}=(f_{b}[{1}]^{T}, \ldots, f_{b}[i]^{T}, \ldots, f_{b}[m]^{T})\in \mathbb {R}^{n\times m}$. Here, $f_{b}[i]^{T}\in \mathbb {R}^{n}$ denotes the feature vector in channel $i$. Subsequently, $O_{B}$ is input into the neuron set $D$. $Soma(\bullet)$ operation assigns $n \times n$ learnable synaptic weights to the $n$ synapses connected to the $n$ neurons in set $D$. All synaptic weights are stacked into the matrix $W_{BD} \in \mathbb {R}^{n \times n}$. Thus, we can calculate the membrane potential vector $v_{d}[i]^{T}\in \mathbb {R}^{n}$ of set $D$ by

\begin{equation*} v_{d}[i]^{T} = -\lambda r_{d}[i]^{T} + W_{BD} f_{b}[i]^{T}, \tag{8}\end{equation*}

where $r_{d}[i]^{T}\in \mathbb {R}^{n}$ denotes the reset membrane potential vector of set $D$. Then, $Soma(\bullet)$ judges whether the membrane potential of the neuron $d_{j}$ in set $D$ satisfies the condition for sending a spiking sequence $x_{d_{j}} = (x_{d_{j}}[{1}], \ldots, x_{d_{j}}[m])^{T}\in \mathbb {R}^{m}$ to the synapse $d_{j}$ via

\begin{equation*} x_{d_{j}} = U(v_{d_{j}} - v_{\textrm {thre}}), \tag{9}\end{equation*}

where $v_{\textrm {thre}}\in \mathbb {R}^{m}$ is an all-ones vector multiplied by the scalar coefficient $V_{\textrm {thre}}$. Afterward, the reset membrane potential $r_{d_{j}}[i]$ in channel $i$ for the neuron $d_{j}$ in set $D$ is updated by taking into account two items: the reset membrane potential $r_{d_{j}}[i-1]$ in channel $i-1$ and the output $x_{d_{j}}[i]$ of neuron $d_{j}$ in channel $i$. The reset process is formulated by

\begin{equation*} r_{d_{j}}[i] = e^{\frac {-1}{\tau _{r}}}r_{d_{j}}[i-1] + V_{\textrm {rest}} x_{d_{j}}[i], \tag{10}\end{equation*}

where $r_{d_{j}}[{0}]$ is initialized as zero and $V_{\textrm {rest}}$ is a scalar coefficient. After completing the calculations in $Soma$, we can get the output $X_{D}=(x_{d_{1}}, \ldots, x_{d_{j}}, \ldots, x_{d_{n}})^{T}\in \mathbb {R}^{n\times m}$ of set $D$, where $x_{d_{j}}\in \mathbb {R}^{m}$ is the output channel sequence of neuron $d_{j}$ in set $B$. When $X_{D}$ passes through the second $Axon(\bullet)$, we can obtain the output matrix $O_{D}=Axon(X_{D})$.

For Path-2, after the input $X_{B}$ passes through the single $Axon(\bullet)$, we can obtain the output matrix $O_{B'}=Axon(X_{B})$. After completing all the calculations along both pathways, we can acquire the output of $F_{2}(\bullet)$ block as $O_{S}= F_{2}(X_{B}) = O_{B'} + O_{D}$, which will be input into the set $A$.

#### 3) Fractal-SNN Block

We simulate the neural connection structure in the brain using $F_{2}(\bullet)$ block in a concise manner. However, the brain contains a vast number of neurons that are connected in complex ways. Inspired by assumed fractal structures of brain [23], we further expand the structure of $F_{2}(\bullet)$ by a new fractal rule. Actually, fractal structures have been widely used in the networks [24], [25]. These studies validate the potential value of fractals in enhancing the representational power and performance of neural networks.

**FIGURE 5.**
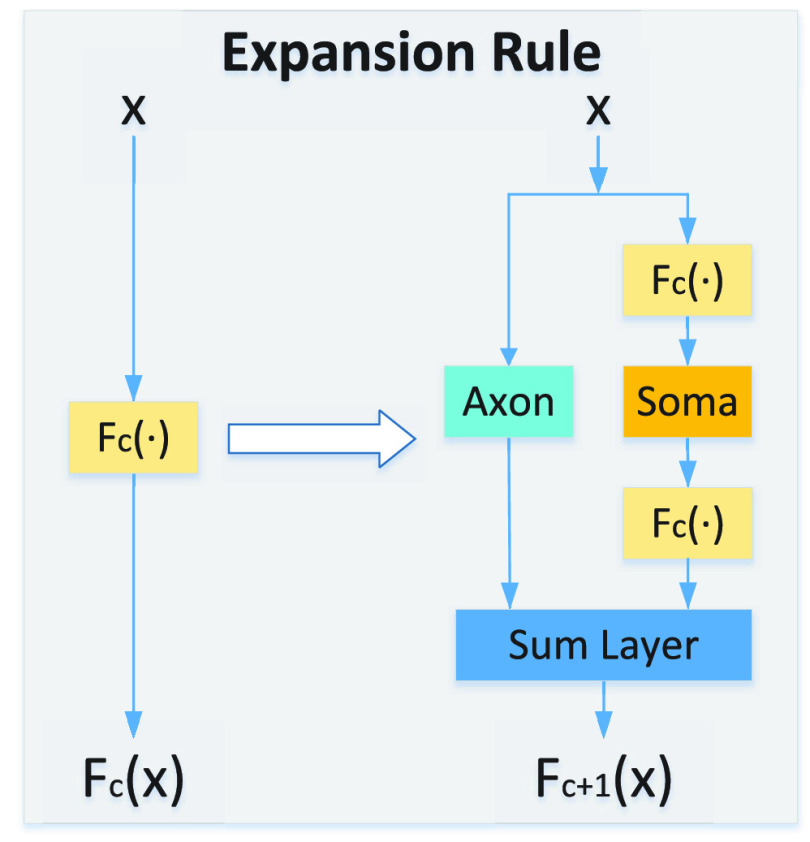
Expansion rule of the Fractal-SNN block.

Let $F_{c}(\bullet)$ be the Fractal-SNN block, then the expansion rule of the Fractal-SNN block can be illustrated in Fig. 5, in which $F_{c}(X)$ and $F_{c+1}(X)$ are the structures of the $c^{\textrm {th}}$ and $(c+1)^{\textrm {th}}$ iterations of the fractal block, respectively. The new expansion rule is defined as

\begin{align*} F_{1}(X) &= Axon(X), \tag{11}\\ F_{c+1} (X)&= Sum\left \{{(F_{c}\circ Soma\circ F_{c}) (X), Axon(X)}\right \}, \tag{12}\end{align*}

where $X$ and $F(X)$ denote the input and output of the Fractal-SNN block, respectively; $Sum\{\bullet, \bullet \}$ means the element-wise sum; $\circ $ represents the function composition. According to this fractal rule, we can easily infer that the $F_{2}(\bullet)$ block is actually a model generated by this rule, especially for the case of c = 2. The instantiation of $F_{3}(\bullet)$ has been illustrated in Fig. 1. In greater detail, we have described the forward calculation process of $F_{2}(\bullet)$ block. Without loss of generality, the output of $F_{2}(\bullet)$ can be extended to the output of $F_{c}(\bullet)$.

#### 4) Inverted Drop-Path Technique

Inspired by the inverted drop-out technique (i.e., a variant of the dropout technique [26]) and the drop-path technique [24], we design a new inverted drop-path technique for the Fractal-SNN block to enhance its generalization ability. This technique works at the $Sum~Layer$ of the fractal block in the training phase. The inverted drop-path technique randomly deactivates part of sub-paths at $Sum~Layer$ with a certain probability $p$, and multiplies the output of each active path with $1/(1-p)$. With the inverted drop-path technique, scaling is applied in the training phase, but not applied in the testing phase. With a normal drop-path technique in the testing phase, the actual value of output is larger than the expectation in training. Therefore, the inverted drop-path technique is beneficial for improving the generalization performance of the Fractal-SNN block. Suppose that there are a total of $k$ paths in the Fractal-SNN block, the input from the $c^{\textrm {k}}$ path to the $Sum~Layer$ is $O^{c_{k}}$, and the inverted drop-path rate is $p= P(\Phi =0)$, in which the random variable $\Phi $ satisfies the Bernoulli distribution and $P$ denotes the probability for $\phi =0$. Thus, the output $O$ of $Sum~Layer$ is

\begin{equation*} O = Sum\{\Phi O^{c_{1}}, \ldots, \Phi O^{c_{k}}\} / (1-p). \tag{13}\end{equation*}

#### 5) Mechanism of Fractal-SNN Block

The inverted drop-path technique, through the random deactivation of a portion of the path during the training phase, allows for the training of various sub-networks within the Fractal-SNN block in training instances. These sub-networks exhibit paths of different lengths and possess varying degrees of feature representation capability. Upon completion of the training process, these sub-networks collaborate to exploit the multi-scale TSS information of the data. Therefore, the Fractal-SNN block with a group of long and short paths implicitly benefits from the following mechanism: the deep and shallow sub-networks can cooperate with each other to adaptively exploit the complementary deep and shallow TSS information from the complicated data, which is conducive to the balance of discrimination and generalization abilities of the learned block.

### E. Dimensionality Reduction and Classification

The inputs $X\in \mathbb {R}^{n\times m}$ learned by the Fractal-SNN block form the Fractal-SNN representation, e.g., $F_{c}(X)\in \mathbb {R}^{n\times m}$, where $n$ is the feature vector dimension and $m$ is the signal channel number. Then, the data flow after the Fractal-SNN block is as follows. Firstly, the Fractal-SNN representation flows into $Soma(\bullet)$ to produce the output spike matrix $Y_{o}\in \mathbb {R}^{n_{1}\times m}$. Next, the spike matrix $Y_{o}$ is fed into a linear layer, a leaky ReLU layer, and another linear layer to reduce the dimensionality of the features. Specifically, the first linear layer increases the dimensionality of features of $Y_{o}$ to generate $Y_{o1}\in \mathbb {R}^{2n_{1}\times m}$, which helps alleviate the loss of information caused by the non-linear activation function [27]. The second linear layer reduces the dimensionality of the features of $Y_{o1}$ to generate $Y_{o2}\in \mathbb {R}^{z\times m}$, where $z$ denotes the number of categories to be classified. Afterward, a final linear layer weights and sums the logits over all channels of $Y_{o2}$, resulting in the prediction vector $y_{p}\in \mathbb {R}^{z}$. Finally, this vector is converted into a probability distribution through the softmax layer for final classification.

## III. Experiments

### A. Experimental Setup

We evaluate our proposed Fractal-SNN scheme on four public benchmark databases, DREAMER [14], DEAP [15], SEED-IV [16], and MPED [17]. DREAMER contains 14-channel EEG signals. During data acquisition, 23 subjects were watching 18 film clips. After this, the subjects rated evaluation from 1 to 5 in the dimension of valence, arousal, and dominance. DEAP contains 32-channel EEG signals. During data acquisition, 32 subjects were watching 40 excerpts of music videos. After this, the subjects gave evaluation values from 1 to 9 in the dimension of valence, arousal, and dominance. Further, we separate the emotion indicators into two classes for valence, arousal and dominance, respectively. Specifically, we utilize the middle scale as the threshold for labeling: if the scale is not less than the threshold, the class label is set as “high”, otherwise “low”. And, the middle scale is 3 for DREAMER and 5 for DEAP. SEED-IV contains 62-channel EEG signals from 15 subjects while they were watching 24 Chinese films. The films were labeled for 4 discrete emotions (i.e., neutral, sad, fear, and happy). MPED contains 62-channel EEG signals collected from 23 subjects while they were watching 28 movies. The movies were labeled for 7 discrete emotions (i.e., joy, funny, anger, fear, disgust, sad and neutrality).

Two groups of experiments are conducted in this study: one for the recognition of continuous emotions, such as arousal and valence, and the other for the recognition of multiple discrete emotions, such as happy, fear, and sad. DREAMER and DEAP databases are utilized to evaluate the performance of the proposed scheme for classifying of continuous emotions, while SEED-IV and MPED databases are employed to assess the performance of the scheme for classifying discrete emotions.

For DREAMER and DEAP, considering the validity of emotional information in EEG data, we use the signals captured in the last-60-second of each film clip, which has also been recommended by the database establishers [14], [15]. The evaluation protocols for Subject-Dependent (SD) emotion recognition are described in the following. On DREAMER, we adopt the 9-fold cross-validation protocol: for the 18 samples of each subject, 16 samples are used as the training data while the remaining 2 samples as the testing data per fold, and the cross-validation results are further averaged among all the 23 subjects as the final performance; on DEAP, we adopt the 10-fold cross-validation protocol: for the 40 samples of each subject, 36 samples are used as the training data while the remaining 4 samples as the testing data per fold, and the cross-validation results are further averaged among all the 32 subjects as the final results. The evaluation protocols for Subject-Independent (SI) emotion recognition are described in the following. We adopt the Leave-One-Subject-Out (LOSO) cross-validation protocol for both DREAMER and DEAP: all samples from one subject are used as the testing data while from the remaining subjects as the training data in turn, and the validation results are further averaged among all the subjects as the final performance.

For SEED-IV and MPED, we directly use the EEG features provided by databases. The SD protocols are adopted in the experiments. To be more specific, on SEED-IV, we follow the experimental settings in the original article [16] to use the first 16 trials for training and the remaining 8 trials that contain all emotions for testing. On MPED, we adhere to Protocol 3 in the SD mode specified by the database establisher [17] to divide the training and testing sets: 21 trials are used for training and the remaining 7 trials containing all emotions are used for testing.

All the experiments are conducted on the computer with CPU of AMD Ryzen 7 5800X 8-Core Processor, GPU of NVIDIA GeForce RTX 3080 Ti, and RAM of 32 GB. In experiments, the Fractal-SNN scheme adopts the parameter settings in Table 1 by default, except those specified.

**TABLE 1:** Default Parameter Settings of the Fractal-SNN Scheme

Parameters	DREAMER	DEAP	SEED-IV	MPED
$Feat$	PSD	PE, DE	DE	PSD
$\tau _{m}$	4	4	4	4
$\tau _{s}$	1	1	1	1
$\lambda $	1	1	1	1
$V_{\text {thre}}$	0	0	0	0
$V_{\text {rest}}$	0.5	0.5	0.5	0.5
$c$	3	3, 2	2	2
$h$	1, 4	1, 4	5	5
$n_{1}$	512	512	500	500
$p$	0.15	0.15	0.15	0.15
$lr$	1e-4	2e-2, 1e-4	1e-4, 2e-3	1e-4
$wd$	1e-2	1e-4	1e-2, 1e-3	1e-2
$Opt$	AdamW	SGD, AdamW	Adam, AdamW	AdamW

Notes: Feat means the features of the input; PE represents Preprocessed EEG; PSD represents Power Spectral Density; DE represents Differential Entropy; $\tau_m, \tau_s, \lambda, V_{\text {thre }}$ and $V_{\text {rest }}$ are the parameters of spiking neurons; $c$ denotes the fractal coefficient of $F_c(\bullet) ; h$ denotes the number of heads in multi-head self-attention; $n_1$ denotes the neuron number of Soma following $F_c(\bullet)$ in Fig. 1; $p$ denotes the drop-path rate; $l r$ denotes the learning rate; $w d$ denotes the weight decay; $O p t$ means the optimizer.

### B. Feature Evaluation

We compare the performance of our scheme with four feature extraction methods, including PE, Temporal Encoding (TE) [28], PSD and DE under the SD protocols on DREAMER and DEAP. These methods convert EEG signals into various forms of features, which enhance the hidden information in the original signals from different perspectives. a) The PE signals are the electrical signals expressed in the form of time series, and the mean value of the signals is guaranteed to be zero after preprocessing; b) TE features preserve the information such as the time position, duration, and sequence of EEG signals; c) PSD and DE features are more effective in reflecting the periodicity and frequency distribution in data. If the EEG signal is given to the model without undergoing any preprocessing, the amplitude of the input signal that is usually too large can present challenges in selecting an appropriate spiking neuron threshold. Without an appropriate threshold, a significant number of spiking neurons may become deactivated or activated during the training process, making it difficult to optimize the model effectively. The results have been shown in Table 2. From the results on DREAMER, we can see that PSD features are beneficial for our proposed scheme to acquire a superior performance compared to the other features. From the results on DEAP, we can find that PE signals are conducive to our scheme to achieve an advantageous performance. By contrast, the proposed scheme performs poorly based on the TE features on both databases.

**TABLE 2:** Evaluation on the Features for the Fractal-SNN Scheme on DREAMER and DEAP (Accuracy / %)

Databases	Features	Valence	Arousal	Dominance
DREAMER	PE	68.36	74.64	78.50
	TE	57.97	68.12	74.64
	PSD	71.01	78.50	80.92
	DE	70.53	75.85	77.78
DEAP	PE	69.84	69.61	73.20
	TE	60.44	58.04	66.52
	PSD	65.16	69.22	70.55
	DE	62.27	68.43	68.87

Abbreviations: Temporal Encoding - TE.

### C. Threshold Selection for Spiking Neurons

**FIGURE 6.**
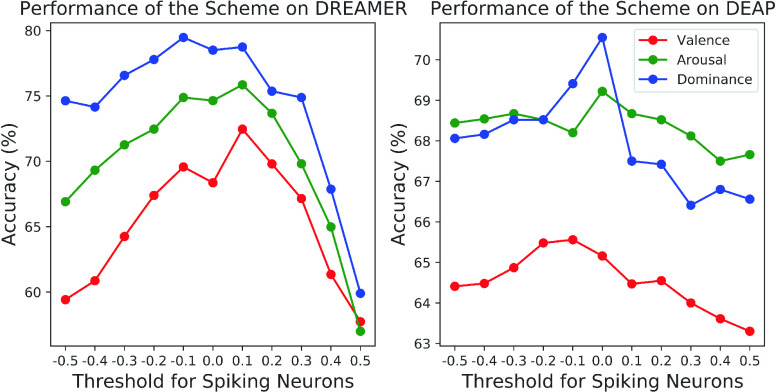
Performance of the Fractal-SNN scheme with different thresholds for spiking neurons on DREAMER and DEAP.

We investigate the impact of threshold $V_{\textrm {thre}}$ for spiking neurons on the performance of our scheme under the SD protocol on DREAMER and DEAP, when using PSD features as the input data. As shown in Fig. 6, when the threshold $V_{\textrm {thre}}$ increases from −0.5 to 0.5, we can notice that all the performances of our scheme for valence classification, arousal classification and dominance classification exhibit a general trend on the two databases: the accuracy first increases and then decreases. The reason behind this phenomenon is as follows: when the threshold is too small, the spiking neurons are easily to be activated, but the discrimination ability of the model is weakened; when the threshold is too large, the spiking neurons are difficult to be activated, and too many inactivated neurons incline to cause a decline of model classification performance. Furthermore, there exists small difference between the performance trends on DREAMER and DEAP. This difference stems from the inherent discrepancy of data distributions between these two databases. Even so, when the threshold $V_{\textrm {thre}}$ is around 0, our scheme can consistently obtain relatively high performances on both databases. Therefore, we recommend setting the threshold $V_{\textrm {thre}}$ to a value around 0.

### D. Fractal Architecture Evaluation

At first, we evaluate the effect of the Fractal-SNN block with different fractal structures on the performance of the proposed scheme under the SD protocols on DREAMER and DEAP, when using the PE as the input data. The results have been provided in Table 3, from which we can see $F_{3}(\bullet)$ is a better choice than $F_{1}(\bullet)$ and $F_{2}(\bullet)$; and, when using $F_{4}(\bullet)$ as the Fractal-SNN block, the performance is degraded to a certain extent compared with using $F_{3}(\bullet)$. Moreover, $F_{3}(\bullet)$ block retains both $F_{1}(\bullet)$ and $F_{2}(\bullet)$ structures and does not have so complicated structure as $F_{4}(\bullet)$. The above results show that appropriately increasing the complexity of the fractal block structure is beneficial to enhancing the scheme performance, but an over complex structure tends to bring about overfitting for the scheme.

**TABLE 3:** Evaluation on the Fractal-SNN Block With Different Structures on DREAMER and DEAP (Accuracy / %)

Databases	Fractal Structures	Scheme Depth	Valence	Arousal	Dominance
DREAMER	$F_{1}(\bullet)$	9	64.73	73.67	74.64
	$F_{2}(\bullet)$	12	64.73	74.15	76.09
	$F_{3}(\bullet)$	18	68.36	74.64	78.50
	$F_{4}(\bullet)$	27	67.87	74.15	76.09
DEAP	$F_{1}(\bullet)$	9	62.03	68.28	68.52
	$F_{2}(\bullet)$	12	64.69	69.37	70.16
	$F_{3}(\bullet)$	18	69.84	69.61	73.20
	$F_{4}(\bullet)$	27	65.08	68.28	66.65

**FIGURE 7.**
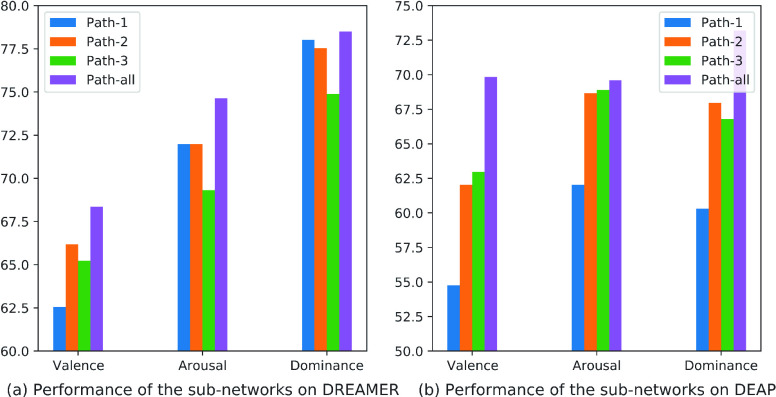
Evaluation on the sub-networks in the Fractal-SNN block on DREAMER and DEAP (Accuracy / %).

Then, we evaluate the sub-networks with only one path in the $F_{3}(\bullet)$ block and the block itself with all the paths. Here we test three situations: Path-1, Path-2, and Path-3, which respectively correspond to the three paths in the $F_{3}\left ({\bullet }\right)$ block instantiation given in Fig 1. The results have been shown in Fig. 7. From the results, we can see that the Fractal-SNN block with all the paths performs better than one single path on both databases. In greater detail, it is difficult to summarize a fixed relationship between the performances of sub-network and the “length” of paths. The main reason is as follows. On the one hand, the long-path sub-network has advantage in learning the deep, detailed and discriminative features from signals; such kinds of features are usually strong in the classification ability but probably weak in the generalization ability. On the other hand, the short-path sub-network has advantage in learning the robust, rough and representative features from signals; such kinds of features are probably weak in the classification ability but usually strong in the generalization ability. An ideal feature learning block should be good at both abilities. However, in practice, which ability is more important for enhancing the sub-network performance also depends on the actual conditions of task objective and data distribution. Even so, the consistent superiority of the Fractal-SNN block with all paths in different conditions manifests that its fractal architecture is able to well balance both classification and generalization abilities in EEG-based emotion recognition.

### E. Inverted Drop-Path Technique Evaluation

We evaluate the performance of the Fractal-SNN scheme with different drop-path techniques under the SD protocols on DREAMER and DEAP, when using the PE as the input data. By default, the Fractal-SNN block is instantiated as $F_{3}(\bullet)$, and the inverted drop-path and original drop-path rate are set to 0.15. The results have been provided in Table 4. It can be seen from the results that, on both databases, the Fractal-SNN scheme employing the inverted drop-path technique yields superior performance compared to utilizing the drop-path one, which also outperforms the case without using any drop-path strategy. This is because the proposed scheme trained without any drop-path strategy is easy to incur overfitting, but the scheme trained with the inverted drop-path technique obviously improves such a phenomenon. In other words, the inverted drop-path technology can effectively enhance the generalization ability of our scheme.

**TABLE 4:** Evaluation on the Fractal-SNN Block Learned by Different Drop-Path Techniques on DREAMER and DEAP (Accuracy / %)

Databases	Training Techniques	Valence	Arousal	Dominance
DREAMER	None Drop-Path	61.59	69.81	74.88
	Drop-Path	66.18	70.29	76.81
	Inverted Drop-Path	68.36	74.64	78.50
DEAP	None Drop-Path	63.19	67.89	68.99
	Drop-Path	63.59	69.29	68.52
	Inverted Drop-Path	69.84	69.61	73.20

### F. Method Ablation

#### 1) Ablation on Multi-Head Attention Module

We carry out an ablation study on the multi-head attention module in the Fractal-SNN scheme under the SD protocol on DREAMER. The goal of this study is to evaluate the contribution of this module to the performance of our scheme and probe into how different settings of this module influences the performance of the whole scheme. In the ablation study, we evaluate three variants of our scheme with different configurations of the attention module. The first variant is the scheme without any attention module; the second variant is the scheme with the one-head attention module; the third variant is the scheme with the four-head attention module.

The results presented in Table 5 indicate that the scheme with one single attention head has achieved the highest classification performance in terms of both valence and arousal indicators, with an accuracy rate of 71.01% and 78.50%, respectively. The scheme with four attention heads has achieved the best classification performance in terms of the dominance indicator, with an accuracy rate of 81.88%. When the attention module is disabled, the scheme shows the poorest performance. Based on these results, we can conclude that the multi-head attention module plays an important role in our proposed scheme for EEG-based emotion recognition.

**TABLE 5:** Ablation of the Multi-Head Attention Module on DREAMER (Accuracy / %)

Ablation Variants	Features	Valence	Arousal	Dominance
no head	PSD	70.29	75.84	76.33
	DE	69.57	73.91	76.33
one head	PSD	71.01	78.50	80.92
	DE	70.53	75.85	77.78
four heads	PSD	70.53	77.54	81.88
	DE	70.29	73.91	76.33

#### 2) Ablation on Fractal-SNN Block

**FIGURE 8.**
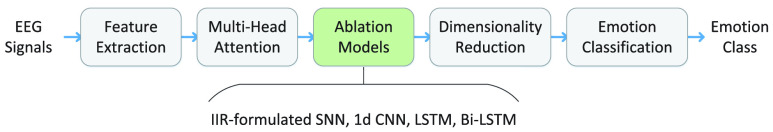
Different neural networks available to substitute for the Fractal-SNN block in the proposed scheme.

To evaluate the effectiveness of the Fractal-SNN block, we conduct an ablation study to evaluate overall performance of the proposed block under the SD protocol on DREAMER, when using PSD features as the input data. As depicted in Fig. 8, the Fractal-SNN block in the architecture can be replaced by different neural networks, including the IIR-formulated SNN, one-dimensional CNN (1d-CNN), LSTM and bidirectional LSTM (Bi-LSTM). These results have been presented in Table 6. The results clearly show that the Fractal-SNN block surpasses the performance of the other alternatives by a significant margin, which provide strong evidence for the effectiveness of the design of the Fractal-SNN block.

**TABLE 6:** Ablation of the Fractal-SNN Block on DREAMER (Accuracy / %)

Ablation Models	Valence	Arousal	Dominance
IIR-formulated SNN	66.18	71.98	77.54
1d-CNN	59.66	65.46	68.84
LSTM	66.91	73.19	77.78
Bi-LSTM	65.70	72.71	78.02
Our scheme	71.01	78.50	80.92

### G. Signal Channel Discussion

**FIGURE 9.**
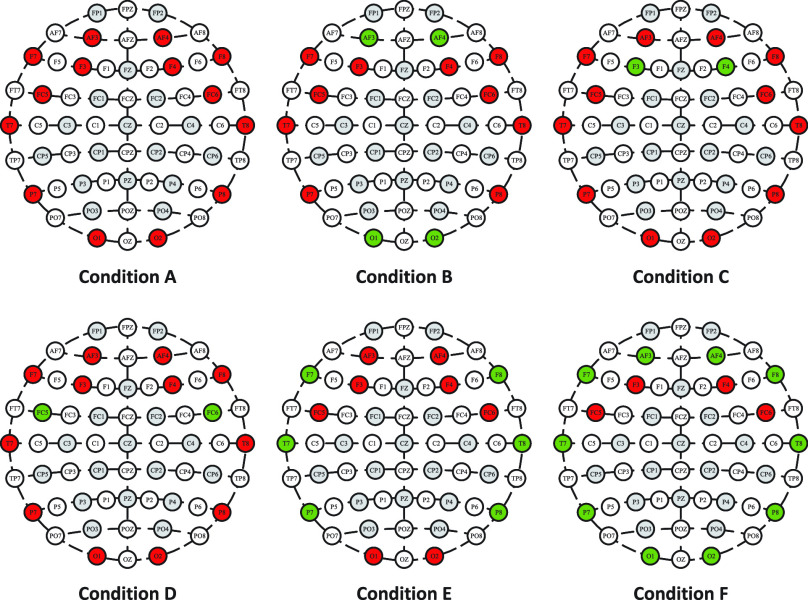
EEG channel conditions (the green nodes represent the disabled channels, and the red nodes represent the used channels) on DREAMER.

We evaluate the performance of Fractal-SNN scheme based on different choices and combinations of EEG signal channels under the SD protocol on DREAMER, when using PE as the input data. For this database, the electrode layout follows the International 10-20 system, where the electrodes are placed symmetrically. So, we disable different portions of signal channels in a symmetric manner to evaluate the Fractal-SNN scheme. The six conditions of disabled signal channels have been visualized in Fig. 9. The experimental results have been given in Table 7. From the results, we can observe that when all the signal channels are used (i.e., Condition A), the proposed scheme performs the best. This is mainly because more channels can bring more data resources for our scheme to exploit the useful information. Moreover, when the number of used channels is reduced with a certain limit (i.e., Conditions B to E), the scheme accuracy fluctuates slightly overall, which in some extent reflects the robustness of our scheme to the disability of signal channels. Furthermore, when much fewer channels are used (i.e., Condition F), the scheme performance declines dramatically. Unsurprisingly, in this condition, insufficiency of the available sample data unavoidably impedes the scheme performance into full play.

**TABLE 7:** Evaluation on the Fractal-SNN Scheme Under Different Conditions of Signal Channels on DREAMER (Accuracy / %)

Conditions	Unused Channels	Valence	Arousal	Dominance
A	–	68.36	74.64	78.50
B	AF3, AF4, O1, O2	67.63	71.26	76.09
C	F3, F4	66.91	70.98	75.85
D	FC5, FC6	66.67	71.01	75.60
E	F7, F8, T7, T8, P7, P8	67.63	71.74	76.09
F	AF3, AF4, O1, O2, F 7, F8, T7, T8, P7, P8	63.77	70.53	72.71

### H. Interpretability Analysis

We take advantage of saliency mapping to highlight the regions of the input features that have the largest contributions to the performance of the Fractal-SNN scheme for discrete emotion classification under the SD protocol. The 62-channel features from SEED-IV [16] have been utilized for experiments: DE in five frequency bands ( $\delta, \theta, \alpha, \beta $ and $\gamma $). The feature vectors of these five frequency bands are further concatenated into a feature matrix $X^{\textrm {feat}}\in \mathbb {R}^{n\times m}$, where $n=500$ is the number of features and $m=62$ is the number of channels. Specifically, the index intervals of the features of the $\delta $, $\theta $, $\alpha $, $\beta $ and $\gamma $ bands are [0, 100), [100, 200), [200, 300), [300, 400), and [400, 500), respectively. Here, we randomly select subject-8 from this database to illustrate the saliency maps, without loss of generality.

**FIGURE 10.**
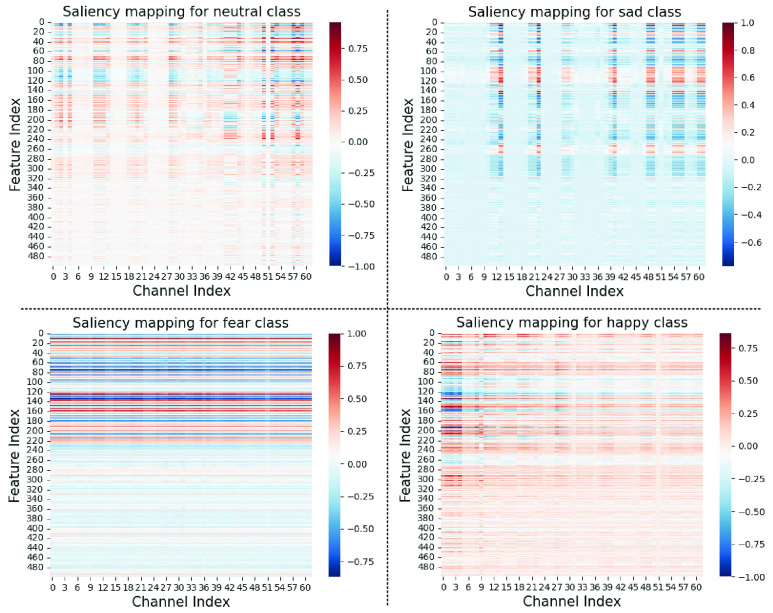
Saliency mapping of the Fractal-SNN scheme on subject-8 from SEED-IV.

To generate the saliency maps, we normalize the feature matrix of the selected subject and deliver the data into the Fractal-SNN scheme at first, and then backpropagate the output classification scores to the input and calculate the gradient. Finally, we normalize the gradient and generate the saliency maps for four discrete emotions: neutral, sad, fear and happy. In Fig. 10, the maps display that the Fractal-SNN scheme is able to capture the TSS features discriminative for different emotions. More concretely, for the fear emotion, signal bands $\delta $ and $\theta $ oscillate in almost all the channels, while, for the happy state, the oscillations of signal bands $\delta $, $\theta $, $\alpha $ and $\beta $ from channel-0 to channel-5 are relatively stronger. Obviously, different emotions have caused different oscillations of the frequency bands of signals. These results readily demonstrate the effectiveness of the Fractal-SNN scheme in capturing the emotion-discriminative information from EEG.

### I. Reliability Analysis

**FIGURE 11.**
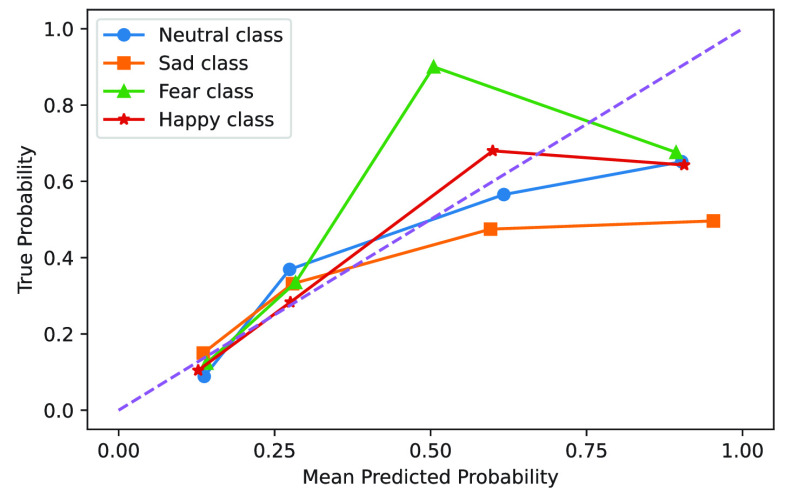
Reliability diagram of the Fractal-SNN scheme on SEED-IV (the dotted line indicates the perfect calibration).

We evaluate the reliability of the Fractal-SNN scheme under the SD protocol on SEED-IV by the reliability diagram in Fig. 11. For every emotion class, we calculate the mean predicted probability of all the testing samples in each of the four interval bins, including [0, 0.25), [0.25, 0.5), [0.5, 0.75), and [0.75, 1.0]; at the same time, we calculate the true probability of the samples correctly classified in each bin [29].

Fig. 11 shows that, for most bins, the mean predicted probability is close to the true one for the proposed scheme. Meanwhile, we also observe that the actual calibration of our scheme has more or less deviations from the perfect one. In greater detail, for the neutral, sad and happy classes, the mean predicted probability in the range between 0 and 0.75 is close to the true one for our scheme. Such results indicate the reliability of our scheme in this probabilistic range for class prediction. However, our scheme tends to overestimate the probability in the range between 0.75 and 1.0 for predicting the neutral, sad and happy classes. As for the fear class, the class prediction of our scheme is more reliable in the probabilistic range between 0 and 0.5. But, the scheme seems over-conservative for the samples with the mean predicted probability between 0.5 and 0.75 and over-confident for those between 0.75 and 1.0. On the whole, these results indicate the overall reliability of the Fractal-SNN scheme for four-class emotion prediction of most data samples.

### J. Method Comparison

#### 1) Results on Dreamer and DEAP

**FIGURE 12.**
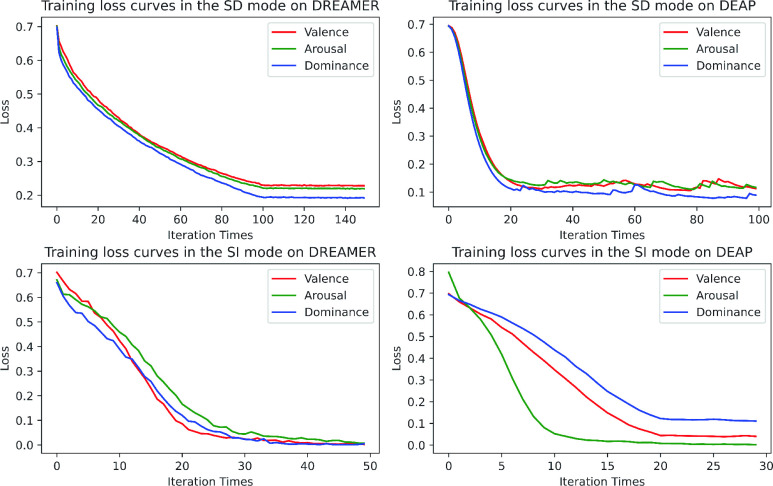
Training loss curves of the Fractal-SNN scheme on DREAMER and DEAP.

We compare our proposed scheme with the related methods under the SD and SI protocols on DREAMER and DEAP. The compared methods include [3], [5], [6], [10], [13], [30], [31], [32], [33]. It is worth mentioning that some of the compared methods have been evaluated in different ways in their original papers. For fairness, we implement all the methods under the same evaluation protocols in this work. The training loss curves of Fractal-SNN have been displayed in Fig. 12.

From the results on DREAMER in Table 8, we can find that the Fractal-SNN scheme has acquired remarkable performances: the accuracy of 71.01% in the SD mode for valence classification, 78.50% in the SD mode and 70.53% in the SI mode for arousal classification, and 80.92% in the SD mode and 75.12% in the SI mode for dominance classification. Moreover, the proposed scheme with the core of the Fractal-SNN block has outperformed the other two SNN-based models, i.e., IIR-formulated SNN and Neucube-based NeuroSense. Further, our scheme has outperformed all other related methods in the SD mode. All these results have validated the advantage of our Fractal-SNN scheme for EEG-based emotion recognition, particularly in the SD mode.

**TABLE 8:** Comparison of the Fractal-SNN Scheme With Related Advanced Methods on DREAMER and DEAP (Accuracy / %)

Databases	Methods	Models	Valence	Arousal	Dominance	Protocols
DREAMER	Li et al., 2019 [30]	FCN	62.15	53.86	67.50	SD (9)-Fold)
			60.63	62.46	57.05	SI (LOSO)
	Li et al., 2020 [31]	JDA-NN	66.50	58.27	68.64	SD (9)-Fold)
			60.55	65.03	63.26	SI (LOSO)
	Fang et al., 2021 [10]	IIR-formulated SNN	57.02	65.32	72.13	SD (9)-Fold)
			54.03	62.32	64.25	SI (LOSO)
	Tan et al., 2021 [13]	Neucube-based NeuroSense	57.00	65.22	69.08	SD (9)-Fold)
			52.90	61.60	63.77	SI (LOSO)
	Li et al., 2021 [3]	BiDANN	65.14	64.17	67.07	SD (9)-Fold)
			59.98	63.26	65.36	SI (LOSO)
	Zhong et al., 2022 [5]	RGNN	54.40	59.42	63.02	SD (9)-Fold)
			54.68	60.23	55.92	SI (LOSO)
	Tuncer et al., 2022 [32]	Tetromino Pattern+SVM	59.90	57.25	53.64	SD (9)-Fold)
			$\textbf {67.15}^{\ddagger} $	69.80	67.39	SI (LOSO)
	Li et al., 2022 [6]	BiSMSM	65.45	66.43	76.23	SD (9)-Fold)
			60.86	63.28	72.71	SI (LOSO)
	Ours	Fractal-SNN Scheme	$\textbf {71.01}^{\#}$	$\textbf {78.50}^{\#}$	$\textbf {80.92}^{\#}$	SD (9)-Fold)
			57.49	$\textbf {70.53}^{\ddagger} $	$\textbf {75.12}^{\ddagger} $	SI (LOSO)
DEAP	Li et al., 2019 [30]	FCN	45.55	62.73	59.06	SD (10)-Fold)
			53.63	55.55	60.31	SI (LOSO)
	Li et al., 2020 [31]	JDA-NN	54.21	52.70	56.06	SD (10)-Fold)
			52.44	54.40	59.26	SI (LOSO)
	Fang et al., 2021 [10]	IIR-formulated SNN	62.66	67.66	68.52	SD (10)-Fold)
			51.20	53.90	56.52	SI (LOSO)
	Tan et al., 2021 [13]	Neucube-based NeuroSense	58.44	62.34	58.44	SD (10)-Fold)
			* $\textbf {67.76}^{\ddagger} $	* $\textbf {78.97}^{\ddagger} $	–	SI (LOSO)
	Li et al., 2021 [3]	BiDANN	54.48	56.92	60.94	SD (10)-Fold)
			53.34	54.40	56.25	SI (LOSO)
	Zhong et al., 2022 [5]	RGNN	50.12	51.34	56.32	SD (10)-Fold)
			49.69	51.88	51.48	SI (LOSO)
	Tuncer et al., 2022 [32]	Tetromino Pattern+SVM	57.73	64.77	62.13	SD (10)-Fold)
			57.27	56.64	60.94	SI (LOSO)
	Li et al., 2023 [33]	TARDGCN	57.73	58.12	63.02	SD (10)-Fold)
			57.73	$\textbf {58.35}^{\ddagger} $	$\textbf {61.69}^{\ddagger} $	SI (LOSO)
	Ours	Fractal-SNN Scheme	$\textbf {69.84}^{\#}$	$\textbf {69.61}^{\#}$	$\textbf {73.20}^{\#}$	SD (10)-Fold)
			$\textbf {60.70}^{\ddagger} $	58.04	61.25	SI (LOSO)

Abbreviations: Functional Connectivity Network - FCN, Joint Distribution Adaptation with Neural Network - JDA-NN, Infinite Impulse Response - IIR, Spiking Neural Network - SNN, Bi-hemisphere Domain Adversarial Neural Network - BiDANN, Regularized Graph Neural Network - RGNN, Bi-Stream MLP-SA Mixer - BiSMSM, Support Vector Machine - SVM, Trainable Adjacency Relation Driven Graph Convolutional Network - TARDGCN, Subject Dependent - SD, Subject Independent - SI, Leave-One-Subject-Out - LOSO.

Notes:
1. * indicates that the method is implemented based on the experimental protocols and train-test data splits in its original paper; 2. – indicates that the authors of the original paper did not conduct the corresponding experiment; 3. # indicates the best performance in the SD mode; 4. ‡ indicates the best performance in the SI mode.

From the results on DEAP in Table 8, we can see that the Fractal-SNN scheme has achieved the best results on the whole: the accuracy of 69.84% in the SD mode and 60.70% in the SI mode for valence classification, 69.61% in the SD mode for arousal classification, and 73.20% in the SD mode for dominance classification. As a special case, we may notice that Neucube-based NeuroSense [13] has obtained the best performance for valence and arousal classification in the SI mode on DEAP. Since the computational cost of NeuroSense in this mode is so high that the out-of-memory problem inevitably occurs for a normal PC with 32-GB RAM, we directly report the result of this method from its original paper here. Nevertheless, we are concerned that the excessive memory consumption may greatly limit the method practicability in real applications. Moreover, there seems no comparability for the reported high accuracy of NeuroSense in the SI mode, because of its different experimental setting from ours. But our scheme still outstrips by a large margin under the same evaluation protocol in the SD mode. Furthermore, the proposed scheme with the core of the Fractal-SNN block performs the best among all comparison methods in the SD mode. However, we also notice that the performance of our scheme in the SI mode is lower than that in the SD mode overall, which is mainly caused by the data distribution discrepancy between different individuals. On the whole, these results have confirmed the advantage of our Fractal-SNN scheme for SD EEG-based emotion recognition again.

#### 2) Results on Seed-Iv and MPED

**FIGURE 13.**
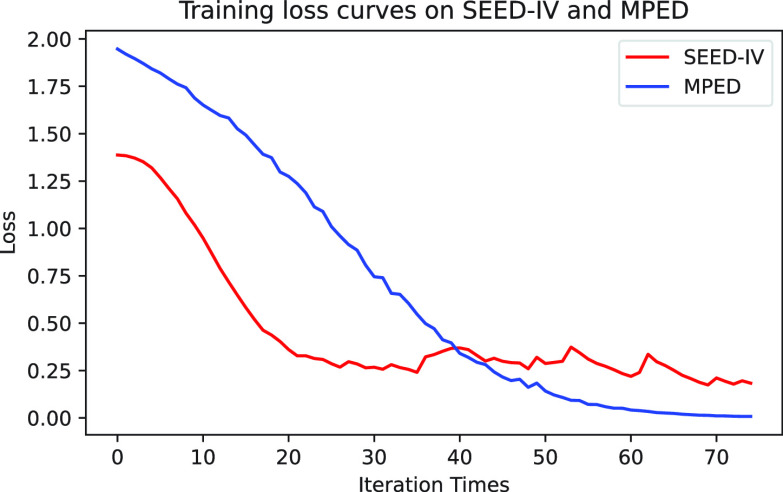
Training loss curves of the Fractal-SNN scheme on SEED-IV and MPED.

We compare our proposed scheme with the related methods under the SD protocols on SEED-IV and MPED. The compared methods include [5], [6], [10], [13], [17], [32], [34], [35], [36], [37]. In order to ensure the fairness of performance comparison among different methods, a unified data division way mentioned in Section III-A is adopted for all the methods. The training loss curves of Fractal-SNN have been displayed in Fig. 13. As can be observed from the results in Table 9, the accuracy of the Fractal-SNN scheme surpasses the compared SNN models [10], [13] and all the other rivals on SEED-IV and MPED. Specifically, our proposed scheme has obtained an accuracy of 68.33% on SEED-IV and 42.23% on MPED. These results readily confirm the capability of our proposed scheme for multi-class emotion recognition based on EEG.

**TABLE 9:** Comparison of the Fractal-SNN Scheme With the Related Advanced Methods on SEED-IV and MPED (Accuracy / %)

Databases	Methods	Accuracy
SEED-IV (Four-Class Classification)	IIR-formulated SNN, 2021 [10]	51.67
	Neucube-based NeuroSense, 2021 [13]	45.00
	RGNN, 2022 [5]	56.39
	SFFM, 2022 [34]	51.13
	Tetromino Pattern+SVM, 2022 [32]	34.38
	BiSMSM, 2022 [6]	58.90
	Ours	68.33
MPED (Seven-Class Classification)	A-LSTM, 2019 [17]	38.43
	IIR-formulated SNN, 2021 [10]	32.30
	Neucube-based NeuroSense, 2021 [13]	28.57
	GMSS, 2021 [35]	40.11
	GECNN, 2022 [36]	40.98
	V-IAG, 2023 [37]	40.40
	Ours	42.23

Abbreviations: Saliency based Feature Fusion Model - SFFM, attentionLSTM - A-LSTM, Graph based Multi task Self Supervised - GMSS, Graph-Embedded Convolutional Neural Network - GECNN, Variational Instance-Adaptive Graph - V-IAG.

## IV. Conclusion

In this paper, we have proposed a new and effective Fractal-SNN scheme, which can well take advantage of the useful multi-scale TSS information, for the issue of EEG-based emotion recognition. Besides, we have devised the inverted drop-path technique for scheme training to enhance its generalization ability. The experimental results on DREAMER, DEAP, SEED-IV and MPED have demonstrated the suitability and capability of the proposed scheme for EEG-based emotion recognition, especially in the SD mode. Moreover, the bionic idea contained in Fractal-SNN may hopefully shed light on the innovation of more SNN-relevant models for this issue and broader related topics in the future.
